# Influence of blueberry tissue type, wounding and cultivar on susceptibility to infection by *Neofusicoccum* species

**DOI:** 10.1111/jam.15493

**Published:** 2022-02-25

**Authors:** K. M. Shanika Tennakoon, Hayley J. Ridgway, Marlene V. Jaspers, E. Eirian Jones

**Affiliations:** ^1^ Department of Pest‐management and Conservation, Faculty of Agriculture and Life Sciences Lincoln University Lincoln New Zealand; ^2^ Ministry for Primary Industries, Biosecurity New Zealand Wellington New Zealand; ^3^ Present address: The New Zealand Institute for Plant & Food Research Ltd Christchurch New Zealand

**Keywords:** Botryosphaeriaceae, highbush, rabbiteye, *Vaccinium ashei*, *Vaccinium corymbosum*, wounding

## Abstract

**Aim:**

Botryosphaeriaceae causing stem blight and dieback of blueberry are important pathogens limiting economic production worldwide. This study investigated the pathogenicity and relative virulence of isolates from the *Neofusicoccum* species commonly associated with blueberries in New Zealand on different tissues and cultivars of blueberries.

**Methods and Results:**

Both wounded and non‐wounded fruit and flower buds and wounded attached soft green and hard green shoots were susceptible to infection by conidia of *Neofusicoccum australe*, *Neofusicoccum parvum* and *Neofusicoccum ribis*. *N. ribis* was generally most virulent, followed by *N. parvum* and then *N. australe*. Inoculation of potting mixture with *N. australe* or *N. ribis* conidia showed that potting mixtures were not a source of inoculum for infection of blueberry roots. Wounded and non‐wounded leaf buds, fruit and wounded soft green shoots and hard green shoots of the different cultivars tested were susceptible to infection by *N. parvum* and *N. ribis*. Whilst the fruit of all cultivars were similarly infected, infection incidence in inoculated leaf buds was lowest in “Blue Bayou” and “Ocean Blue”. Cultivar susceptibility differed when tested on soft green shoots compared with hard green shoots, with shortest lesions developed on “Maru” on soft green shoots, and “Centra Blue” and “Ocean Blue” on hard green shoots.

**Conclusions:**

All tested above‐ground blueberry tissues, including non‐wounded tissue, were susceptible to *Neofusicoccum* spp. All the cultivars assessed were susceptible to infection, although they varied in their relative susceptibility depending on the tissue assessed.

**Significance and impact of the study:**

The potential for non‐wounded tissue to become infected indicate that fungicides may need to be applied to protect all tissue, not just wounds.

## INTRODUCTION

Blueberries are an economically important crop grown commercially worldwide including Australia, Canada, China, Europe, New Zealand, South America and the USA (FAO, 2020). The New Zealand industry has been an active participant in developing blueberries as a global fruit following their domestication in the early 20th century. Currently, there are over 60 growers and 400 ha planted in New Zealand, with most of the plantings in the upper North Island, although new areas are being planted in the South Island (www.blueberriesnz.co.nz). Both *Vaccinium ashei* (rabbiteye) and *V. corymbosum* (highbush) are commercially cultivated. Blueberry dieback caused by members of the family Botryosphaeriaceae are increasingly being recognized as major issues for blueberry production worldwide (Xu et al., 2015). Botryosphaeriaceae infection causes stem blight, dieback, cankers and crown rot, which are estimated to affect 18% of blueberry plants in the main production areas in New Zealand (Sammonds et al., 2009). The species of Botryosphaeriaceae associated with blueberry stem blight are reported to differ between regions, with *Botryosphaeria dothidea, Lasiodiplodia theobromae, Neofusicoccum australe, N. parvum* and *N. ribis* commonly reported as the causal agent (Milholland, 1972; Creswell & Milholland, 1987; Espinoza et al., 2009; Smith, 2009; Wright & Harman, 2010; Castillo et al., 2013; Xu et al., 2015; Borrero et al., 2019; Scarlett et al., 2019; Hilário et al., 2020; Guarnaccia et al., 2020; Rodríguez‐Gálvez et al., 2020; Pečenza et al., 2021). In New Zealand, isolations from necrotic blueberry stems obtained from a survey of blueberry farms identified *N. australe*, *N. luteum*, *N. parvum* and *N. ribis*, with *N. australe* being the most common species recovered (Tennakoon et al., 2018a).

Most pathogenicity studies with blueberries have described the pathogenicity of only one or a few species of Botryosphaeriaceae (Creswell & Milholland, 1987; Espinoza et al., 2009; Milholland, 1972), with very little work having been done to examine the pathogenicity of Botryosphaeriaceae to blueberry in New Zealand. Tennakoon et al. (2018a) conducted a preliminary study by inoculating detached blueberry shoots using mycelial plugs of species of Botryosphaeriaceae. Results showed that isolates of the four main species recovered from farms and nurseries were pathogenic on blueberry stems, but virulence differed significantly between species and isolates within a species, with *N. ribis* being the most virulent, followed by *N. parvum*, *N. luteum* and *N. australe*. Further studies by Tennakoon et al. (2018b) showed that inoculation of wounded green shoots with conidial concentrations of *N. parvum* and *N. ribis* between 10^3^ and 5 × 10^4^ conidia/inoculation site resulted in 100% disease incidence. Infections occurred not only through wounds, but also through non‐wounded tissues.

Blueberry stems and twigs have been shown to be susceptible. However, in grapevines, a wide range of tissues including berries, buds and leaf surfaces were also shown to be susceptible to infection by *N. luteum* (Amponsah et al., 2012a). The susceptibility of different blueberry tissues to the species of Botryosphaeriaceae commonly associated with blueberry in New Zealand is not known and warrants investigation. Further, whether infection can occur through roots from inoculum in propagation media is unknown, especially since various propagation media from commercial nurseries were found to be contaminated with Botryosphaeriaeae (Tennakoon et al., 2018a). Whitelaw‐Weckert et al. (2006) reported infection of grapevines from soil inoculum of *Diplodia mutila*.

The main control strategy for this disease includes removing and destroying infected canes along with fungicide application to protect pruning wounds. However, a more sustainable control strategy would be to use tolerant or resistant cultivars. Critical factors in these screening protocols include stem age, isolate virulence, inoculation environment and timing of evaluation (Smith, 2009). To date, although none of the cultivars tested are reported to be completely resistant to the disease, they are reported to vary in their susceptibility (Smith, 2004). In New Zealand, breeding programs have mainly focused on improvement of yield and fruit quality (Sammonds et al., 2009). The first New Zealand survey by Sammonds et al. (2009) recovered many isolates of different Botryosphaeriaceae from symptomatic tissue of blueberry cultivars “Marimba” and “Elliot”, both of which were confirmed in separate studies in the USA, to be tolerant to *B. dothidea* (Smith, 2009). This indicated the cultivars found to be more tolerant to *B. dothidea* appear to be susceptible to New Zealand isolates of other Botryosphaeriaceae. The relative susceptibility of commonly grown cultivars to the local Botryosphaeriaceae population therefore needs to be determined.

The overall objectives of this study were to determine the pathogenicity of multiple isolates from the Botryosphaeriaceae most commonly associated with blueberries on different tissue types of blueberries, and to determine the susceptibility of different tissues of different cultivars.

## MATERIALS AND METHODS

### Fungal isolates and inoculum production

Three isolates of each of *N. australe* (LUPP1301, LUPP1321, LUPP1364), *N. parvum* (LUPP1249, LUPP1288, LUPP1363) and *N. ribis* (LUPP1300, LUPP1348, LUPP1365) were used. All isolates were originally isolated from blueberry tissue in a New Zealand nationwide survey and stored as mycelial plugs in 20% glycerol at −80°C in the Lincoln University Plant Pathology culture collection (Tennakoon et al., 2018a). The isolates were routinely cultured on potato dextrose agar (PDA; Difco™) at 25°C.

Conidia for inoculation were produced on detached blueberry shoots as described by Tennakoon et al. (2017) and the resulting conidial suspension adjusted to the required concentration based on haemocytometer counts.

## SUSCEPTIBILITY OF DIFFERENT BLUEBERRY TISSUES TO *NEOFUSICOCCUM* SPECIES

### Flower buds and fruit

Two‐year‐old potted plants of rabbiteye blueberry cultivar “Dolce Blue” were used for the experiments. For fruit and bud inoculation, 20–30 fruit/flower bud bunches, depending on availability, selected on each plant were surface sterilized by swabbing with a cotton wool bud and 70% ethanol and allowed to air dry for ~30 s. Of these, 10–20 replicate bunches were wounded and 10–20 replicate bunches were not wounded. Fruits were wounded by pricking (~1 mm deep) 5 times each with a sterile needle which was resterilized between tissue types and plants by dipping in 70% ethanol. The flower buds were individually wounded by scratching lightly with a needle. For each species (*N. australe*, *N. parvum* and *N. ribis*), a mixed isolate conidial suspension adjusted to 10^4^ conidia ml^−1^ containing equal concentrations of each isolate was prepared as described. The selected fruits and buds were each drop inoculated with 20 μl of the mixed conidial suspension of each species or sterile water (control). Each inoculated tissue on a plant was immediately covered with a separate clean transparent plastic bag, misted inside with water and left for 48 h to provide high humidity. Ten replicate plants for each treatment combination were placed in a shadehouse area at the Lincoln University Nursery, in a complete randomized design (CRD) layout. The buds and fruits were observed for any discolouration 10 and 14 days after inoculation, respectively, and removed from the inoculated plants and uninoculated controls. These were surface sterilized in 70% ethanol for 30 s, rinsed with sterile water for 30 s and air dried in a laminar flow cabinet for 10 min. The tissue pieces from the edge of the lesions were plated onto PDA amended with 0.5 g l^−1^ chloramphenicol (PDAC) plates. For the uninoculated control buds and fruit where no lesions developed, representative tissue pieces were surfaced sterilized and plated onto PDAC plates. The plates were incubated under 12 h light and 12 h dark conditions at 25°C and the Botryosphaeriaceae isolates growing onto the agar from the tissue pieces identified by colony appearance as described by Tennakoon et al. (2018a). The proportion of buds and fruit on each of the selected bunches positive for Botryosphaeriaceae isolate infection per replicate plant was used to determine the percentage infection incidence.

### Attached wounded soft green shoots and hard green shoots

Three‐year‐old potted plants of blueberry cultivar “Dolce Blue” were used for the experiments. On each plant, two soft and two hard shoots were surface sterilized by swabbing with 70% ethanol, superficially wounded with a sterile scalpel (~1–2 mm deep and 4–6 mm in diameter) and wrapped with Parafilm™ to form a lip. The sites were immediately inoculated with 50 μl drops of a mixed isolate conidial suspension (10^6^ ml^−1^) of the same species as for the bud and fruit inoculations. Each *Neofusicoccum* species was inoculated onto a different plant and separate plants were used for the uninoculated controls (sterile water). Each plant was covered with a transparent plastic bag sprayed inside with water and this was left in place for 48 h to provide sufficient humidity for infection. Ten replicate plants for each treatment combination were placed in a shadehouse area at the Lincoln University Nursery, in a CRD layout. The shoots were observed for any discolouration and lesion lengths were measured with a digital calliper (Mitutoyo, Kanagawa, Japan), after 14 days for soft green shoots and after 60 days for hard green shoots. The infected tissues were surface sterilized by dipping in 70% ethanol for 30 s and air dried in a laminar flow cabinet for 10 min. Isolations were made onto PDAC using 1 cm stem pieces cut from the lesion edge above and below the inoculation point. After 4 days incubation at 25°C under 12 h light and 12 h dark, colonies were identified by their resemblance to the inoculating isolates.

### *Neofusicoccum* spp. infection of blueberries by potting mix‐root transmission

The experiment was conducted in a commercial nursery in the North Island of New Zealand within their nursery propagation facilities. Two different types of propagation cuttings were used for this experiment: soft green rooted cuttings (cultivar “Blue Bayou”; 10–12 cm long) and 3‐month‐old hard rooted cuttings (cultivar “Blue Bayou”; 10–15 cm long). The hard rooted cuttings were potted in 9.5 × 9.5 cm pots, and the soft green cuttings were potted in each of four cells (5 × 5 cm) of a potting tray, containing commercial soil‐free potting mix. The conidial suspensions (10^4^ ml^−1^) used for inoculation were made from two isolates each of *N. australe* (LUPP1321 and LUPP1364) and *N. ribis* (LUPP1348 and LUPP1365). To ensure good contact between the inoculum and the plants, 250 ml of conidial suspension was poured into a hole (3 cm in depth) made in the potting mix at 2 cm from the stem of each potted hard rooted cutting. For the soft cuttings, 50 ml of inoculum was poured onto the surface of the potting media for each cell in the potting trays. Untreated controls were treated with the equivalent volume of water. Twelve plants were set up for each treatment including the controls, with 12 plants for the rooted hard cuttings, and 3 trays each containing 4 soft cuttings arranged in a CRD. All plants were maintained in the nursery under standard commercial nursery conditions. After 6 months when some plants started to display discolouration, the plants were all sent to the Lincoln University Plant Pathology laboratory. Plants were left under lights (12 h light: 12 h dark) at room temperature, with hand‐watered as required, for 1–2 weeks until they were processed.

For each rooted hard cutting, the stems were separated from the roots and divided into main stem and side shoots. The main stem was surface sterilized by soaking in 70% ethanol for 30 s and air dried under sterile air in a laminar flow hood, and ten 0.5 cm sections were cut from the base, one 0.5 cm section from the middle and one 0.5 cm section from the tip of the main stem. Each section was then separated into bark and wood prior to isolation. From the three sideshoots that grew closest to the base, the first 3 cm was removed and surface sterilized with 70% ethanol prior to cutting into 0.5 cm segments for which bark and wood were separated for isolation. Three tissue segments taken from each of two leaves close to the tip of each plant and three leaf segments from each of three leaves that showed lesions were also plated after undergoing surface sterilization as previously described.

The root systems of each hard rooted cuttings were washed with tap water to remove potting mix and other debris. For each plant, three hard and three soft roots were randomly selected for isolation and surface sterilized by dipping in 20% bleach (sodium hypochlorite 53 g l^−1^) for 3 min, washing with sterile water for 1 min and air drying for 30 min under sterile air in a laminar flow hood. The hard roots were cut into 1 cm pieces for up to 10 cm (depending on length) from the plant base and the three soft roots cut into 1 cm pieces for up to 5 cm from the plant base (depending on length).

For the rooted soft cuttings, the ~10 cm stems were separated from the root system and surface sterilized as described previously and then cut into 0.5 cm segments. Five soft roots were removed from the root system and washed with tap water and then surface sterilized as previously described. Each root was cut into 1 cm pieces, up to 5 cm from the plant base for isolation.

All the plant tissues were plated onto PDAC and the plates were incubated at 25°C in a diurnal light regime (12 h dark: 12 h white light) for 3–5 days. The Botryosphaeriaceae isolates were identified to species level based on morphological identification and molecular identification using amplified ribosomal DNA restriction analysis (ARDRA) as described by Tennakoon et al. (2018a). Mycelial plugs of isolates identified as *N. australe* and *N. ribis* were stored in 20% glycerol at −80°C until extraction of genomic DNA was performed for further genetic analysis, which was used to differentiate the inoculating isolates from naturally occurring isolates.

### Genomic DNA extraction

For DNA extraction, all the isolates recovered from the above experiment and the seven isolates of *N. ribis* (LUPP1274, LUPP1275, LUPP1283, LUPP1287, LUPP1297, LUPP1340 and LUPP1349) used for selection of primers for random amplified polymorphic DNA (RAPD) were grown in potato dextrose broth (PDB; Difco™) and incubated at 25°C in a diurnal light regime (12 h dark: 12 h white light) for 3 days. The mycelium of each isolate was snap frozen in liquid nitrogen and genomic DNA extracted using the PUREGENE® genomic extraction kit (Gentra systems) using the plant tissue DNA isolation protocol according to the manufacturer's instructions. DNA concentrations were measured using a NanoDrop spectrophotometer (Nanodrop Technologies Inc.). All the genomic DNA samples were diluted to working concentrations of 20–25 ng μl^−1^ and stored at −20°C until processed by PCR.

### Selection of primers for RAPD analyses for *N. ribis* isolates

Four RAPD primers, UBC 517 (5′GGTCGCAGCT3′), UBC 598 (5′ACGGGCGCTC3′), UBC 600 (5′GAAGAACCGC3′) and Operon H–‐19 (5′CTGACCAGCC3′), were screened to select the primer most likely to detect polymorphism (Singh & Hughes, 2006). The DNA extracted from the seven isolates listed previously, which were randomly selected from different farms (Tennakoon et al., 2018a) and the two *N. ribis* isolates (LUPP1348 and LUPP1365) used to inoculate the propagation media, were amplified with each of the RAPD primers (Singh & Hughes, 2006).

PCR was conducted as described by Baskarathevan et al. (2011) except that the 20 pmol of UP‐PCR primer was replaced with 10 pmol of an RAPD primer. PCR amplifications were performed in a Veriti Thermal Cycler‐200 as follows: initial denaturation at 94°C for 6 min, followed by 45 cycles of denaturation at 92°C for 1 min, an annealing temperature of 36°C for 1 min and extension at 72°C for 1 min, with a final extension at 72°C for 6 min. All the PCR products were separated by electrophoresis on a 1.3% agarose gel and agarose gels were stained and photographed as described by Tennakoon et al. (2018a).

From the four primers, one primer was selected for the final RAPD analyses. The criteria for the selection of primers were based on the total number of bands amplified, the number of polymorphic bands and how easily they distinguished the *N. ribis* isolates (LUPP1348 and LUPP1365). The RAPD analysis was repeated with the selected primer for all the *N. ribis* isolates recovered from the propagation cuttings. The banding patterns of the inoculating isolates (LUPP1348 and LUPP1365) were compared with all the *N. ribis* isolates recovered to determine if the individuals were genetically similar.

### Susceptibility of fruit and leaf buds of different blueberry cultivars to *N. parvum* and *N. ribis*

Two‐year‐old potted plants of seven blueberry cultivars (“Blue Bayou”, “Centra Blue”, “Dolce Blue”, “Maru”, “Ocean Blue”, “Powderblue” and “Rahi”) were used for the experiments. For fruit inoculations, the 20–30 surface sterilized fruit bunches selected on each plant were either wounded or not wounded (10–20 replicate bunches each depending on availability) as described for fruit previously. The selected fruit were inoculated with the mixed isolate conidial suspension of either *N. parvum* or *N. ribis*, or sterile water (control plants) as previously described. For leaf bud inoculations, 5–10 leaf buds were selected on two shoots each for wounding and non‐wounding treatment. The buds were surface sterilized, wounded and inoculated as described previously for fruit buds. Six replicate plants for each treatment combination (six plants for each species and cultivar) were placed in a shadehouse area at the Lincoln University Nursery, in a CRD layout. Each plant was covered with a transparent plastic bag sprayed with water for the first 48 h as previously described. The buds and fruit were observed for any discolouration and removed from the inoculated and uninoculated control plants, 10 days and 14 days after inoculation, respectively. Based on the visual symptoms, 10 apparently infected wounded and non‐wounded fruit and leaf buds per plant were used for isolation as described previously.

### Susceptibility of soft green shoots and hard green shoots of different blueberry cultivars to *N. parvum* and *N. ribis*

For soft green shoots and hard green shoots, the same cultivars used for the fruit and buds were used. Shoots were wounded and inoculated with a mixed isolate conidial suspension of either *N. parvum* or *N. ribis*, or sterile water (control plants) as described previously. In this experiment, for each species and cultivar, three stems (soft green or hard green) were inoculated per plant. Six replicate plants for each species and cultivar treatment combination were placed in a shadehouse area at the Lincoln University Nursery, in a CRD layout. The plants were covered with a transparent plastic bag sprayed with water for the first 48 h as previously described. The shoots were observed for any discolouration and lesion lengths were measured with a digital calliper (Mitutoyo, Kanagawa, Japan), after 14 days for soft green shoots and after 60 days for hard green shoots. Isolations were carried out from lesion edges on to PDAC as described previously.

### Statistical analysis

Data of lesion lengths and isolation incidences were analysed by analysis of variance (ANOVA) to determine treatment effects. Since no necrotic lesions developed on any of the non‐inoculated control treated tissues, and no *Neofusicoccum* spp. isolates were recovered from these tissues, these were omitted from the analysis. In experiments where two stems were inoculated on the same plant, these were used to calculate a mean value for that replicate and this was used in the analysis. Comparisons between means of individual treatments used Fisher's protected LSDs at *p* ≤ 0.05. Where significant main effects (e.g. species, wounding treatment or cultivar) or interactions were identified, the treatment means were compared using Fisher's protected least significant difference (LSD) tests at *p* ≤ 0.05. All analyses were conducted using Genstat version 16 (VSN International Ltd).

## RESULTS

### Susceptibility of different blueberry tissues to *Neofusicoccum* species flower buds and fruit

On fruit, inoculation by all three *Neofusicoccum* species caused necrotic lesions on wounded berries within 3–6 days and non‐wounded berries within 9–12 days. The infected berries became mummified and produced pycnidia after a further 14–20 days for both wounded and non‐wounded fruit. Infection incidence was significantly affected by species (*p* < 0.001) and by wounding (*p* < 0.001) but not by an interaction between species and wounding (*p* = 0.897). Highest incidence was caused by *N. ribis* with mean percent infection incidence of 76.8% (67.0% and 86.5% for non‐wounded and wounded fruit, respectively) (Table 1). This was followed by *N. parvum*, with mean percent infection incidence of 65.8% (57.0% and 74.5% for non‐wounded and wounded fruit, respectively). The lowest mean percent infection incidence of 54.0% was by *N. australe* (46.0% and 62.0% for non‐wounded and wounded fruit, respectively). Mean incidence was 74.3% for wounded fruit, being significantly higher than for non‐wounded fruit (56.7%).

**TABLE 1:** Infection incidence (%) of non‐wounded and wounded fruit and flower buds of blueberry cultivar “Dolce Blue” inoculated with *Neofusicoccum* species

Species	Fruit			Flower buds		
	Non‐wounded	Wounded	Mean^#^	Non‐wounded	Wounded	Mean^#^
*Neofusicoccum australe*	46.0	62.0	54.0 a	49.5	60.0	57.8 a
*Neofusicoccum parvum*	57.0	74.5	65.8 b	60.0	75.0	67.5 ab
*Neofusicoccum ribis*	67.0	86.5	76.8 c	66.0	83.0	74.5 b
LSD			7.83			9.03

^#^For species effect, values within columns followed by the same letter are not significantly different according to Fisher's protected LSD at *p* = 0.05. Interaction between species × wounding was not significant for fruit (*p* = 0.897) or buds (*p* = 0.965).

For flower buds, inoculation by all three *Neofusicoccum* species caused necrotic light brown discolouration on wounded buds within 2–3 days and non‐wounded buds within 3–5 days. The species effect on infection incidence was significant (*p* = 0.009) as was the wounding effect (*p* < 0.001), but not the interaction between species and wounding (*p* = 0.965). Highest mean incidence was caused by *N. ribis*, with 74.5% (66.0% and 83.0% for non‐wounded and wounded flower buds, respectively), followed by *N. parvum*, with 67.5% (60.0% and 75.0% for non‐wounded and wounded buds, respectively) (Table 1). The lowest mean percent infection incidence was caused by *N. australe* with 57.8% (49.5 and 66.0 for non‐wounded and wounded buds, respectively). Mean incidence was 74.7% for wounded buds being significantly higher than for non‐wounded buds (58.5%).

Fungal colonies characteristic of the inoculated isolates were recovered from the lesion edges of all the inoculated berry and bud tissues. No *Neofusicoccum* spp. isolates were recovered from control tissues on which no necrotic lesions developed.

### Attached wounded soft green shoots and hard green shoots

Inoculation with conidia of all the *Neofusicoccum* species tested produced brown to black lesions along the shoots. The lesions started to develop within 3–4 days in both soft green shoots and hard green shoots. In the uninoculated control, no lesions developed apart from some minor discolouration at the wound site. The lesion lengths were significantly affected by species, both in soft green shoots after 14 days and hard green shoots after 60 days (*p* < 0.001 for both). In both types of shoots, there were no significant differences (*p* > 0.05) in the length of the lesions caused by *N. ribis* and *N. parvum*, which were both significantly (*p* < 0.05) longer than for *N. australe* (Table 2). Fungal colonies characteristic of the inoculating isolates were obtained from the lesion edges of all the inoculated shoots and no colonies were recovered from the control shoots.

**TABLE 2:** Mean lesion lengths caused by conidia of three *Neofusicoccum* species inoculated onto attached, wounded soft green shoots after 14 days and hard green shoots after 60 days in blueberry cultivar “Dolce Blue”

Species	Lesion lengths (mm)	
	Soft green shoots	Hard green shoots
*Neofusicoccum australe*	24.4 a^#^	18.8 a^#^
*Neofusicoccum parvum*	69.4 b	34.5 b
*Neofusicoccum ribis*	90.1 b	41.3 b
LSD	20.95	8.59

^#^Values within columns followed by the same letter are not significantly different according to Fisher's protected LSD at *p* = 0.05.

### *Neofusicoccum* spp. infection of blueberries by potting mix‐root transmission

When the plants were assessed 6 months after inoculation of the potting mixture, some plants started to show discolouration in the leaves, but no lesions or dieback were observed in either the potted rooted soft cuttings or rooted hard cuttings. When the isolations were carried out from different plant tissues, isolates were recovered from some plant tissues of soft rooted cuttings and rooted hard cuttings which were identified by ARDRA as *N. parvum* and *N. ribis*. Of the 12 soft rooted cuttings and 12 rooted hard cuttings for which soil was infested with *N. australe*, no isolates identified as *N. australe* were recovered. For these plants, two *N. parvum* isolates were recovered from the bark (2.5 and 3.0 cm stem sections from the base of the cutting) of one rooted hard cutting and two *N. ribis* isolates from the 3.5 and 4.0 cm stem sections from the base a soft rooted cutting.

For rooted hard cuttings planted in soil infested with *N. ribis*, isolates of *N. ribis* were mostly recovered from the hard roots, soft roots and the base of the plants where they were restricted to the bark. Of the 12 rooted hard cuttings, 8 plants (66.7%) were positive for the presence of *N. ribis*, with a total of 15 isolates recovered. For the soft rooted cuttings, *N. ribis* isolates were recovered from some soft roots (no hard roots had developed) and the soft stems of the plants. Of the 12 rooted soft cuttings, 6 were infected (50.0%) with *N. ribis*, with a total of 12 isolates recovered. In rooted hard cuttings, *N. parvum* (4 isolates) were recovered from the bark of the base of a one plant, being found in segments up to 4 cm continuously in plant no. 4. In soft rooted cuttings planted in soil infested with *N. ribis*, *N parvum* isolates were recovered from the stems of two plants (2.5 cm stem section in one plant and 4.0 cm stem section in another plant). For the controls of hard rooted cuttings, one plant was infected with *N. parvum*, with one isolate being recovered from the bark at the base of the plant.

### Selection of primers for RAPD analyses of *N. ribis* isolate

Of the four primers tested on the representative isolates, all the primers produced multiple bands. Both *N. ribis* isolates (LUPP1348 and LUPP1365) produced unique fingerprints with primer Operon H‐19.

**FIGURE 1:**
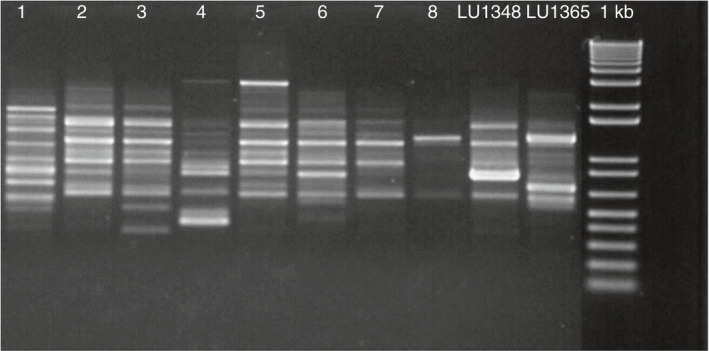
Agarose gel of RAPD amplification with primer operon H–19 of genomic DNA from *Neofusicoccum ribis* isolates recovered from blueberry plants grown in potting mixture infested with conidia of *N. ribis* LUPP1348 and LUPP1365. From left to right, lanes 1–8 isolates recovered from rooted blueberry cuttings. The far‐right lane has the 1 kb plus DNA ladder

Further analysis conducted with primer Operon H‐19 using the isolates recovered from the plants which had been grown in potting mix inoculated with the two *N. ribis* isolates (LUPP1348 and LUPP1365) showed that no isolate had the DNA fingerprint of either isolate LUPP1348 or LUPP1365 (Figure 1).

## SUSCEPTIBILITY OF BLUEBERRY CULTIVARS TO *N. PARVUM* AND *N. RIBIS*

### Fruit and leaf buds

On fruit, both *Neofusicoccum* species caused necrotic lesions on wounded berries within 4–8 days and non‐wounded berries within 10–12 days followed by mummification of the berries and pycnidia development after a further 14–25 days. Infection incidence was not significantly affected by cultivar (*p* = 0.081), although there was a trend that “Blue Bayou” (16.7%) and “Ocean blue” (17.1%) had the lowest infection incidence and “Dolce Blue” (38.7%) had the highest infection incidence (Table 3). Infection incidence was not significantly affected by species (*p* = 0.271). The overall infection incidence for *N. parvum* was 21.7% and for *N. ribis* 26.2%. There was a significant effect of wounding, with overall infection incidence for non‐wounded fruit and wounded fruit being 18.1% and 29.8%, respectively. There was no significant interaction between cultivar, species and wounding (*p* = 0.669).

**TABLE 3:** Mean infection incidence (%) in non‐wounded (NW) and wounded (W) fruit and leaf buds of different blueberry cultivars inoculated with two *Neofusicoccum* species

Cultivar	% infection incidence									
	Fruit					Leaf buds				
	*Neofusicoccum parvum*		*Neofusicoccum ribis*		Mean	*Neofusicoccum parvum*		*Neofusicoccum ribis*		Mean
	NW	W	NW	W		NW	W	NW	W	
“Blue Bayou”	21.7	15.0	6.7	23.3	16.7	8.3	3.3	11.7	13.3	9.2 a^#^
“Centra Blue”	11.7	36.7	31.7	30.0	27.5	8.3	16.7	38.3	56.7	30.0 cd
“Dolce Blue”	21.7	55.0	16.7	61.7	38.7	13.3	38.3	31.7	51.7	33.8 d
“Maru”	15.0	20.0	16.7	38.3	22.5	11.7	31.7	23.3	46.7	28.3 bcd
“Ocean Blue”	15.0	16.7	16.7	20.0	17.1	15.0	11.7	25.0	23.3	18.8 ab
“Powderblue”	16.7	16.7	31.7	25.0	22.5	18.3	6.7	18.3	45.0	22.1 bc
“Rahi”	20.0	21.7	11.7	36.7	22.5	15.0	16.7	40.0	28.3	25.0 bcd
Species mean	17.4	26.0	18.8	33.6		12.8	17.9	26.9	37.9	

^#^Values within the columns followed by the same letter are not significantly different according to Fisher's protected LSD at *p* = 0.05.

On leaf buds, both *Neofusicoccum* species caused necrotic lesions on wounded buds within 3–4 days and on non‐wounded buds within 5–6 days. Infection incidence was significantly affected by cultivar (*p* < 0.001), with the cultivar “Blue Bayou” (9.2%) showing the lowest infection incidence and “Dolce Blue” (38.0%) showing the highest infection incidence (Table 3). The infection incidence was significantly affected by species (*p* < 0.001), with overall infection incidence for *N. parvum* being 15.4% and for *N. ribis* 32.4%. There was a significant effect of wounding, with overall infection incidence for non‐wounded leaf buds and wounded leaf buds being 19.9% and 27.9%, respectively. There was no significant interaction between cultivar, species and wounding (*p* = 0.051). Fungal colonies characteristic of the inoculated isolates were recovered from the lesion edges of all the inoculated berry and leaf bud tissues. No *Neofusicoccum* isolates were recovered from control tissues on which no necrotic lesions developed.

### Soft green shoots and hard green shoots

Inoculated soft green shoots started to develop light brown to reddish lesions along the stems of all the cultivars 3–4 days after inoculation with *N. parvum* and *N. ribis*. There was a significant species effect (*p* < 0.001) over all the cultivars, with longer lesions for *N. ribis* (94.8 mm) than for *N. parvum* (19.6 mm) (Table 4). There was a significant interaction between cultivar and species (*p* < 0.001; Table 5), which was mainly related to the relative susceptibility of “Maru” to *N. parvum* and *N. ribis* compared with the other cultivars. For “Maru”, there was no significant difference in the length of lesions produced by *N. parvum* (26.6 mm) compared with *N. ribis* (36.6 mm). In comparison, for all other cultivars, the lesions produced by *N. ribis* (78.1–149.0 mm) were significantly longer than those produced by *N. parvum* (11.1–24.2 mm). The greatest species difference was observed on “Centra Blue” for which the mean lesion length was 11.1 mm for *N. parvum* and 149.0 mm for *N. ribis*, which were significantly different (Table 4). For *N. parvum*, there was no significant difference in the lesion lengths produced on the different cultivars. In contrast, for *N. ribis*, lesions produced on “Maru” (36.6 mm) were significantly shorter than on all other cultivars, with the lesions produced on “Centra Blue” (149.0 mm) being significantly longer than on all other cultivars. There was also a significant cultivar effect (*p* < 0.001), with the shortest lesion length developing on “Maru” which were significantly shorter than those which developed on all other cultivars apart from “Blue Bayou” and “Ocean Blue”.

**TABLE 4:** Lesion lengths (mm) in soft green shoots of different blueberry cultivars 14 days after inoculation with *Neofusicoccum* species

Species	Cultivar							Species mean
	“Blue Bayou”	“Centra Blue”	“Dolce Blue”	“Maru”	“Ocean Blue”	“Powderblue”	“Rahi”	
*Neofusicoccum parvum*	17.3 ab^#^	11.1 a^z^	24.2 ab	26.6 ab	17.3 ab	19.4 ab	21.5 ab	19.4 x^§^
*Neofusicoccum ribis*	78.1 c	149.0 e	118.3 d	36.6 b	79.2 c	87.3 c	115.4 d	94.8 y
Cultivar mean	47.7 AB^†^	80.1 D	71.3 CD	31.6 A	48.2 AB	53.4 BC	68.5 CD	

For ^#^cultivar × species interactions (a–e), ^§^species effect (*x*–*y*) and ^†^cultivar effect (A–D) mean values followed by the same letter are not significantly different according to Fisher's protected LSD at *p* = 0.05.

**TABLE 5:** Lesion lengths (mm) in hard green shoots of different blueberry cultivars 60 days after inoculation with *Neofusicoccum* species

Species	Cultivar							Species mean
	“Blue Bayou”	“Centra Blue”	“Dolce Blue”	“Maru”	“Ocean blue”	“Powderblue”	“Rahi”	
*Neofusicoccum parvum*	9.8 a^#^	13.0 a	16.3 a	18.7 a	13.5 a	17.6 a	12.8 a	14.5 x^§^
*Neofusicoccum ribis*	39.5 c	29.2 b	63.9 d	30.4 bc	29.2 b	55.9 d	38.4 bc	40.9 y
Cultivar mean	24.8 A^†^	21.1 A	40.1 B	24.5 A	21.3 A	36.8 B	25.6 A	

For ^#^cultivar × species interactions (a–e), ^§^species effect (*x*–*y*) and ^†^cultivar effect (A–D) mean values followed by the same letter are not significantly different according to Fisher's protected LSD at *p* = 0.05.

In hard green shoots, lesions started to develop in all the cultivars 10–12 days after inoculation with *N. parvum* and *N. ribis*. There was a significant interaction between cultivar and species (*p* < 0.001; Table 5), which was mainly related to the relative susceptibility of cultivars to *N. parvum* and *N. ribis*. For *N. parvum* inoculations, there was no significant difference in the mean lesion lengths produced on the different cultivars (9.8–18.7 mm). In contrast, for *N. ribis* inoculation, lesions produced on “Centra Blue” (29.2 mm) and “Ocean Blue” (29.2 mm) were significantly shorter than those produced on all other cultivars, with the lesions produced on “Dolce Blue” (63.9 mm) and “Powderblue” (55.9 mm) being significantly longer than those produced on all other cultivars. There was also a significant species effect (*p* < 0.001) and a significant cultivar effect (*p* < 0.001) on lesion development. Overall, the lesions in hard green shoots inoculated with *N. parvum* were smaller than for *N. ribis*, with mean lesion lengths of 14.5 and 40.9 mm, respectively (Table 5). Across both species, the lesion lengths which developed on inoculated “Blue Bayou”, “Centra Blue”, “Maru”, “Ocean Blue” and “Rahi” were shorter (Table 5).

Fungal colonies characteristic of the inoculated isolates were recovered from the lesion edges of all the inoculated stem tissues. No *Neofusicoccum* isolates were recovered from control tissues on which no necrotic lesions developed.

## DISCUSSION

This is the first study to show the susceptibility of blueberry flower and leaf buds and fruit to infection by *N. australe*, *N. parvum* and *N. ribis*. Additionally, although both wounded and non‐wounded fruit and bud tissues were susceptible, in general higher infection incidences were observed for wounded than non‐wounded tissues. Similar results have been reported for other hosts, with Espinoza et al. (2009) reporting significantly larger lesions developed on wounded compared with non‐wounded apple and kiwifruit fruit when inoculated with *N. arbuti*, *N. australe* and *N. parvum* blueberry isolates. The authors, however, did not test the pathogenicity of these isolates on blueberry fruit, and there are no reported studies related to infection of blueberry reproductive structures by species of Botryosphaeriaceae. In grapevines, Wunderlich et al. (2011) isolated Botryosphaeriaceae pathogens from dormant buds, flowers and berries of grapevines. They also reported that symptomatic berries were soft, oozing juice, covered in mycelial growth and black pycnidia. Steel et al. (2007) also recorded isolation of Botryosphaeriaceae, *Pestalotia* species and *Phomopsis viticola* from grape flowers and berries throughout the growing season, although Botryosphaeriaceae were isolated in low frequency. When Amponsah et al. (2012a) inoculated leaf buds of grapevines with *N. luteum* conidia, most of the infected buds failed to burst although some buds developed into asymptomatic shoots, but with internal discolouration which originated from the inoculated point. Further, they demonstrated that inoculation of wounded fruit resulted in rotting of the fruit followed by formation of pycnidia and pathogen progression into the supporting shoots. When the wounded and non‐wounded fruits and flower buds of blueberries were inoculated in the current studies, most of the buds and fruits developed discolouration and lesions. In the current study, in general *N. ribis* showed highest infection incidence for wounded and non‐wounded tissues, followed by *N. parvum* and *N. australe*. This corresponds to the previous studies with shoot material (Tennakoon et al., 2018a; Tennakoon et al., 2018b).

The pathogenicity assay using conidia on attached soft green and hard green shoots showed that the time taken for the symptom development was greater for the attached than detached shoots. This is similar to the previous results of Tennakoon et al. (2018a) whereby lesions of 63.1 mm were observed 7 days after inoculation in detached soft green shoots and 61.3 mm after 14 days in attached soft green shoots. For detached hard green shoots, lesions of 20.3 mm length developed after 10 days, whereas in attached hard green shoots, lesions of 31.5 mm developed after 60 days. This is likely to be due to plant defence mechanisms being more active in attached shoots than detached shoots, and has been suggested to be due to the absence of systemic signalling pathways in detached plant material (Eshraghi et al., 2014; Liu et al., 2007). Amponsah et al. (2012b) also reported that *Botryosphaeria* lesions took 10 days to develop on detached grapevine shoots and 60 days on attached shoots. Espinoza et al. (2009) reported similar effects on blueberries; two isolates of *N. parvum* inoculated as mycelial agar plugs produced shorter lesions in attached shoots of “O'Neal” cultivar (13.5 and 20.3 mm lesions within 25 days) and produced longer lesions in detached shoots of the same cultivar (63.9 and 70.7 mm lesions within 25 days). Therefore, the results of pathogenicity studies using detached shoots need to be interpreted with caution as it is likely to not provide an accurate picture of the susceptibility, as they are physiologically very different from attached shoots.

During the survey of Botryosphaeriaceae associated with blueberries conducted by Tennakoon et al. (2018a), nursery propagation material was shown to be infected by these species. Therefore, an experiment was conducted using conidia of *N. australe* and *N. ribis* to investigate whether the propagation mixtures could act as a source of inoculum to infect plants via the roots. After 3 months growth in the inoculated soil‐free potting mixture, 50% of the rooted soft cuttings were infected with *N. ribis* but none were infected with *N. australe*. Most of the isolates were recovered from the shoot tissues, including one *N. parvum* isolate that was probably due to earlier contamination of the shoot used. In hard rooted cuttings, no infection occurred with *N. australe* but 66.7% of cuttings were positive for the presence of *N. ribis* isolates. Comparison of the RAPD PCR genotypes (primer Operon H‐19) of the recovered isolates with that of the inoculating isolates (LUPP1348 and LUPP1365) showed none of the isolates recovered from the plants had the fingerprints of isolates LUPP1348 and LUPP1365. This showed that the isolates recovered from the plants were not the isolates added to the propagation mixtures; thus, inocula in propagation mixtures were not the sources of infection. Most of the isolates were recovered from the soft roots, hard roots and the bases of the plants. Some isolates were recovered from the stem sections 3.5 and 4.0 cm from the base of the plants, which suggested that either the planting material used was already infected or infection from other inoculum sources in the commercial nursery occurred during the experimental period, as these plants did not show the expected continuous isolation from the base. A similar experiment was conducted by Whitelaw‐Weckert et al. (2006), whereby *D. mutila*, applied to soil as mycelial and conidial inoculum, was recovered from the basal end of 3‐year‐old potted Pinot noir vines. The isolates they recovered could have been from splash‐dispersed inoculum from the soil during watering of the plants as their isolations did not show continuous infection from the roots. They concluded that *D. mutila* infections could be initiated by the soil‐borne inoculum, as did Castillo‐Pando et al. (2001) who isolated *D. seriata* from grapevine roots. However, no genotyping of the recovered isolates was carried out in these studies, so the source of these infections cannot be confirmed. In contrast to these studies and similar to the current study, Amponsah et al. (2012a) demonstrated that wounded roots of grapevines were not infected by mycelial or conidial inoculum of *N. luteum*, *N. australe*, *N. parvum* or *D. mutila* when isolations were made after 3 months.

All seven blueberry cultivars were susceptible to *N. parvum* and *N. ribis* to some extent for all the tissue types tested, with infection incidence being 100% in both soft and hard green shoot tissues. However, infection incidence in leaf buds and lesion development in soft green shoots and hard green shoots differed significantly between cultivars and *Neofusicoccum* species. In leaf buds, infection incidence was lowest in “Blue Bayou”, followed by “Ocean Blue”, and in fruit all cultivars were similarly infected. In these tissues, the non‐wounded ones were also infected and developed symptoms after inoculation with *N. parvum* and *N. ribis*. In general, shortest lesions were caused on soft green shoots with cultivar “Maru”, whilst cultivars “Centra Blue” and “Ocean Blue” had shortest lesions in hard green shoots. The infection levels of inoculated fruit in the first experiment only using “Dolce Blue” were overall higher than that observed in “Dolce Blue” plants in the cultivar experiment. The reason for this is unclear as the same isolates, inoculation method and inoculum concentration were used for both experiments. However, the two experiments were conducted at slightly different times of year, being in Feb for the first experiment and Dec for the second cultivar experiment, both of which incubated in the shadehouse, which may have affected the relative susceptibility of the tissue.

In the current study, six rabbiteye (*Vaccinium ashei*) cultivars and one highbush (*V. corymbosum*) cultivar (“Blue Bayou”) were used for pathogenicity studies. The selection was based on the prevalence of rabbiteye cultivars in commercial New Zealand blueberry plantations. In the USA, Polashock and Kramer (2006) evaluated disease progression caused by *B. dothidea* (as *F. aesculi*) as mycelium disc inoculum on 50 blueberry cultivars, which included half‐highbush, lowbush, highbush, southern highbush and rabbiteye cultivars. Half‐highbush cultivars produced the shortest lesions followed by lowbush and highbush cultivars, with the southern highbush and rabbiteye cultivars had the longest lesions. It is not possible to compare between highbush and rabbit eye in the current study as only one highbush was included; however, the mean lesion length which developed on highbush cultivar “Blue Bayou” did not differ in length to those on rabbiteye cultivars “Ocean Blue” and “Maru” on soft or hard green shoots. Milholland (1972) reported significant differences in susceptibility of ten commercial highbush cultivars and eight rabbiteye cultivars evaluated by inoculating green shoots with mycelial plugs of six different *B. dothidea*, with highbush cultivars generally being highly susceptible. Similarly, Creswell and Milholland (1987) reported that of six blueberry cultivars, high bush cultivars were more susceptible than rabbiteye cultivars to stem infection by *B. dothidea* isolates.

The current study with potted plants has shown that all tested above‐ground parts of blueberries were susceptible to *Neofusicoccum* spp. infections. It also demonstrated that *N. ribis* was generally most virulent followed by *N. parvum* and then *N. australe*. *Neofusicoccum ribis* and *N. parvum* were also able to infect non‐wounded flower buds, fruit and leaf buds, which may potentially lead to infection spreading into supporting stems as was shown for grapevines (Amponsah et al., 2012a). Previous studies have also shown that wounding was not necessary for infection of shoots, whereby colonization of the bark led to infection of the underlying woody stem tissue (Tennakoon et al., 2018b). It is therefore clear that in blueberries these *Neofusicoccum* spp. are not just wound pathogens as has been shown for other crops indicating that fungicides may need to be applied to protect all tissue, not just wounds, as previously believed. Fungicides such as carbendazim and tebuconazole have previously been shown to reduce infection of both wounded and unwounded in potted and field blueberry plants (Teenakoon et al., 2019) and have potential to protect tissue from infection from external inoculum. Further, these tissues develop pycnidia and release conidia, therefore providing inoculum in the field for further infections leading to dieback which is the main form of damage recognized by growers. Removing inoculum in the blueberry fields will therefore be vital as infection can take place in the absence of wounding whenever spores are produced, which for grapevines has been shown to be year round (Amponsah et al., 2009). The study also confirmed that *Neofusicoccum* spp. inoculum contaminating the propagation media did not result in root or subsequent plant infection; therefore, ensuring the use of blueberry plant material free of *Neofusicoccum* spp. contamination for nursery propagation will help to ensure the pathogen is not introduced into blueberry farms via contaminated planting material.

## CONFLICT OF INTEREST

None of the authors declare a conflict of interest, with all authors consenting to publication.
